# Muscovy duck reovirus p10.8 protein localizes to the nucleus via a nonconventional nuclear localization signal

**DOI:** 10.1186/1743-422X-11-37

**Published:** 2014-02-24

**Authors:** Dongchun Guo, Na Qiu, Wulin Shaozhou, Xiaofei Bai, Yilong He, Qingshan Zhang, Jian Zhao, Ming Liu, Yun Zhang

**Affiliations:** 1 State Key Laboratory of Veterinary Biotechnology, Harbin Veterinary Research Institute of Chinese Academy of Agricultural Sciences, Harbin 150001, P R China

**Keywords:** Muscovy duck reocvirus, p10.8 protein, Nuclear localization signal

## Abstract

**Background:**

It was previously report that the first open reading frame of Muscovy duck reocvirus S4 gene encodes a 95-amino-acid protein, designed p10.8, which has no sequence similarity to other known proteins. Its amino acid sequence offers no clues about its function.

**Results:**

Subcellular localization and nuclear import signal of p10.8 were characterized. We found that p10.8 protein localizes to the nucleus of infected and transfected cells, suggesting that p10.8 nuclear localization is not facilitated by viral infection or any other viral protein. A functional non-canonical nuclear localization signal (NLS) for p10.8 was identified and mapped to N-terminus residues 1–40. The NLS has the ability to retarget a large cytoplasmic protein to the nucleus.

**Conclusions:**

p10.8 imported into the nucleus might via a nonconventional signal nuclear signal.

## Background

The Muscovy duck reovirus (MDRV) is a member of the *Orthoreovirus* genus, of the Reoviridae family. MDRV is an important poultry pathogen that causes high morbidity and mortality in ducklings. Its genome consisting of 10 segments of double-stranded RNA [1-4], each of which is mono-cistronic, with the exception of the S4 gene, which encodes two proteins in overlapping open reading frames (ORFs).

A regular consequence of viral infection is perturbation of host cell nuclear functions. Although reovirus replication occurs in the cytoplasm, infection could disrupt a variety of host cell nuclear functions, resulting in a virus-induced cytopathic effect in infected cells and tissue injury in the infected host. Both mammalian reovirus σ1s (σ1ns) and avian reovirus p17 localizes to the nucleus in infected and transfected cells [5-7]. Mammalian reovirus σ1s and MDRV p10.8 have been confirmed to induce apoptosis in vivo and in vitro, respectively [8,9], suggesting that they are functionally related.

When we initiated this study, little is known about the activity or properties of the duck reovirus p10.8 protein. Furthermore, this polypeptide has no significant sequence similarity to other known proteins, so its amino acid sequence offers no clues about its function. On the other hand, the fact that the p10.8 is conserved in every Muscovy duck reovirus S4 gene sequence reported so far suggests that p10.8 plays an important function in virus-host interactions. The results of this study demonstrate that p10.8 is a nuclear targeting protein, utilizing a previously unrecognized NLS. This sub-cellular localization studies may shed new light on the potential roles of this proteins in pathogenesis.

## Results

### P10.8 localizes to the nucleoplasm of S14 infected cells

**Figure 1:**
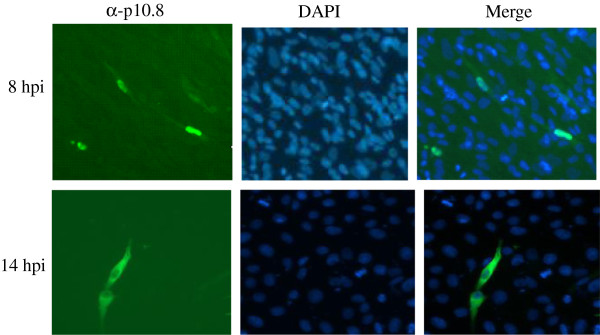
**Localization of p10.8 in S14-infected DEF cells at different hours post infection (hpi).** Infected cells were stained with anti-p10.8 serum and then with an FITC-conjugated goat anti-mouse antibody, and finally with DAPI. Stained cells were visualized by means of fluorescence microscopy (magnification 300×).

The anti-p10.8 antiserum was used to evaluate the subcellular distribution of p10.8 in MDRV S14-infected cells by indirect immunofluorescence. MDRV S14-infected cells were stained at 8 hours post infection (hpi) with antibodies against both p10.8 and then with DAPI. Examinations of the stained cells by means of fluorescence microscopy at 8 hours post-infection (hpi) showed that p10.8 was concentrated within the nucleus (Figure 1 up row). Visualization of infected cells by microscopy also suggested that p10.8 was distributed within the nucleus but not in nucleoli. As infection progressed, p10.8-associated staining cells showed that p10.8 began to accumulate in the cytoplasm of infected cells. Figure 1 (down row) showed that p10.8 was mainly located in the cytoplasm at 14 hpi. On the other hand, p10.8 nuclear targeting was not dependent on the host cell types, since p10.8 protein exhibited a robust nuclear signal both in S14-infected DEF and Vero cells (data not shown). This result indicated that p10.8 might be able to locate in the nucleus of infected cells.

### P10.8 nuclear localization is independent on viral infection

**Figure 2:**
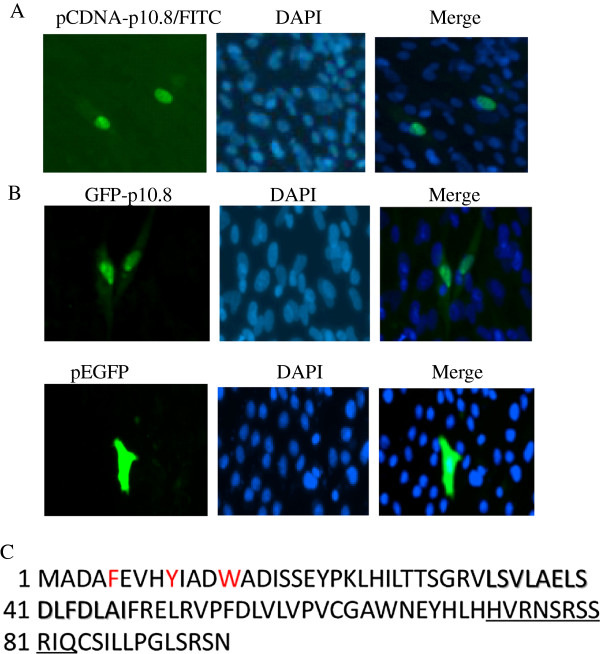
**Transfection of DEF with different plasmids and amino acid sequence analysis of p10.8 protein. (A)** Confluent monolayers of DEF cells were transfected with the pCDNA-p10.8 plasmid. The cells were fixed at 7 hpt, and then stained with serum against p10.8 and with DAPI. **(B)** Confluent monolayers of Vero cells were transfected with pEGFP/pEGFP-p10.8. The cells were fixed at 7 hpt and then stained with DAPI. Stained cells were visualized by use of fluorescence microscopy (magnification 400). **(C)** Deduced amino acid sequence of duck reovirus p10.8 protein. The basic putative NLS is underlined and the putative NES is shaded. Aromatic amino acids are in red.

To determine whether the intranuclear location of p10.8 was dependent on viral factors and/or viral infection, we next examined the intracellular distribution of p10.8 in transfected cells. Confluent Vero or DEF cells were transfected with 5 μg of the recombinant pCDNA-p10.8 plasmid by using the FuGENE HD transfection reagent (Roche Applied Science) and were then immunostained with the mouse anti-p10.8 serum and with DAPI, as described above. Cells were visualized by means of fluorescence microscopy, which revealed that p10.8 accumulated in the nucleus of the transfected DEF (Figure 2A) and Vero cells (data not shown) at 7 h post-transfection (hpt), confirming that p10.8 nuclear targeting was not dependent on cell type or viral factors. Subsequently, cells were transfected with GFP-p10.8 or pEGFP-C1 and then stained with DAPI. Visualization of the cells by means of fluorescence microscopy revealed that GFP-p10.8 accumulated in the nucleus of the transfected cells at 7 hpt (Figure 2B up row), whereas GFP alone was distributed evenly between the nuclear and cytoplasmic compartments (Figure 2B down row). These results demonstrate that p10.8 can target appended GFP to the nucleus, indicating that GFP fusion proteins may be used for further studies as described previously [6].

### Mapping p10.8 nuclear localization functional sequence

**Figure 3:**
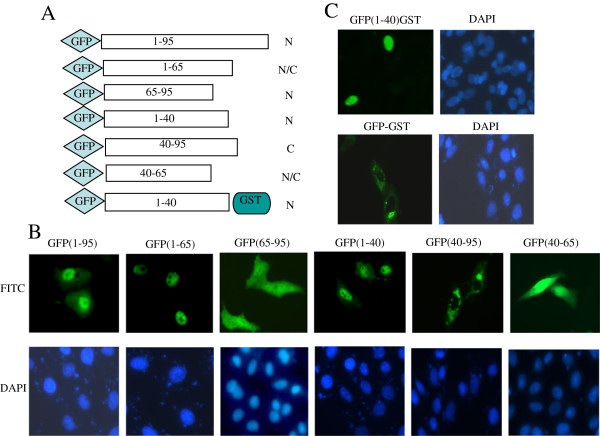
**Intracellular distribution of p10.8 and truncated p10.8 fragments. (A)** Schematic representation of C- and N-terminal deletion mutants of p10.8. **(B)** Cells transfected with the indicated GFP p10.8 fragments plasmids were analyzed by fluorescence microscopy (magnification 400×) (up row) and stained with DAPI (down row). **(C)** Cells transfected with GFP(1–40)GST (up row) and GFP-GST (down row) plasmids were analyzed for GFP expression by fluorescence microscopy and stained with DAPI.

Most nuclear proteins enter the nucleus through specific interactions between the nuclear import machinery and specific protein sequences known as nuclear localization signals (NLSs). NLSs have been shown to contain a continuous motif of basic amino acid residues [10,11]; to define the NLS responsible for p10.8 nuclear import, we scanned the p10.8 sequence and notice a cluster of basic amino acids, which may serve as NLS(73HV**R**NS**R**SS**R**IQ83) (basic amino acids underlined, Figure 2C). To evaluate the potential nuclear import function of this motif, we generated a series of deletion constructs (see Figure 3A). As a first approach to assess whether this basic region represents a functional NLS, we evaluated the nuclear targeting capacity of two p10.8 fragments comprising the first 65 or the last 30 residues of the p10.8 protein. For this experiment, constructs with GFP fused to the N- or C–terminus of p10.8 or to the p10.8 regions spanning residues 1 to 65 [named GFP(1–65)] and 65 to 95 [named GFP(65–95)] were transfected into cells and the subcellular localization of the fused proteins was monitored by use of fluorescence microscopy. Similar to the full-length p10.8 protein, GFP(1–65) localized to the nucleus of the transfected cells (Figure 3B). Conversely, like GFP alone, GFP(65–95) was distributed in both the nucleus and the cytoplasm (Figure 3B), suggesting that GFP(65–95) is not a nuclear protein and that the basic region 73HV**R**NS**R**SS**R**IQ83 is not a functional NLS. We, therefore, constructed N- and C–terminal deletion mutants (amino acids 1–40, 40–95, and 40–65) to define the sequences involved in the nuclear localization. We found that the first 40 amino acids of p10.8 are indispensable for nuclear localization, because deletion mutant GFP(40–95) located exclusively in the cytoplasm and GFP(40–65) distributed evenly in both the nucleus and the cytoplasm of cells, like GFP alone (Figure 3B). Thus, we mapped the potential nuclear localization functional region of p10.8 within residues 1– 40.

### Residues 1 to 40 with the ability to translocate GST protein to the nucleus

Because p10.8 has a mass of 10.8 kDa, it may be that GFPp10.8(1–40) (less than 60 kDa) entered the nucleus by passive diffusion. To ensure that the nuclear localization of GFPp10.8(1–40) was dependent upon active p10.8 signal-mediated nuclear import and was not the result of passive diffusion, we expressed GFPp10.8(1–40) as a fusion with the cytoplasmic reporter protein (glutathione *S*-transferase, GST) (55 kDa). The large size of the GFPp10.8(1–40)-GST fusion protein (about 93 kDa) ensured that the nuclear localization was not due to passive diffusion. Vero cells were transfected with either GFP-GST or GFP-p10.8(1–40)-GST and analyzed via fluorescence microscopy. As expected, the GFP-GST construct that lacked p10.8(1–40) was restricted to the cytoplasm (Figure 3C); however, insertion of p10.8 (1–40) resulted in the translocation of GFPp10.8(1–40)-GST to the nucleus (Figure 3C), indicating that p10.8(1–40) was able to direct GST into the nucleus through the NPC by a signal-mediated import mechanism and not by passive diffusion, since the molecular mass of GFPp10.8GST (93 kDa) exceeded the 50- to 60-kDa limit for free diffusion through NPCs. From these experiments, we conclude that amino acids 1–40 are necessary for the nuclear localization of a heterologous protein that is otherwise cytoplasmic.

**Figure 4:**
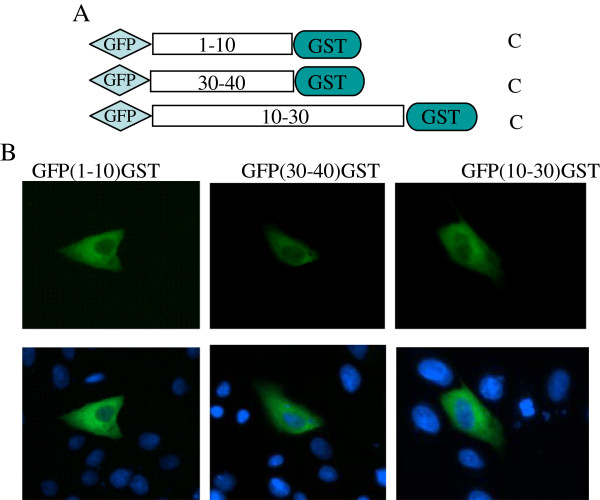
**Intracellular distribution of truncated GFP(1–10)GST, GFP(30–40)GST, and GFP(10–30)GST fragments. (A)** Schematic representation of C-/N-terminal and deletion mutants of p10.8 (1–40). **(B)** Cells transfected with the indicated fragments plasmids were analyzed for GFP expression by fluorescence microscopy (magnification 400×).

To determine whether a few additional amino acids at the amino and carboxyl ends of p10.8(1–40) may be removable without the loss of nuclear localization, we further evaluate the potential nuclear import function motif. We generated a series of deletion constructs (see Figure 4A). For this experiment, constructs with GFP-GST fused to the N- or C-terminus of p10.8 (1–40) or to the region spanning residues 10 to 30 [named GFP(10–30)] were transfected into cells and the subcellular localization of the fused proteins was monitored by use of fluorescence microscopy. Similar to the GFP(40–95), constructs GFP(1–10), GFP(10–30), and GFP(30–40) all localized to the cytoplasm of the transfected cells (Figure 4B), suggesting that these three regions are indispensable for nuclear import function.

## Discussion

This study demonstrates that p10.8 targets the nucleus of transfected cells and that this targeting does not require other viral factors, thereby suggesting that the viral protein itself has the ability to translocate across the nuclear pore complex (NPC) and accumulate in the nucleus. Because p10.8 has a mass of ~10.8 kDa, it was possible that it might enter the nucleus by passive diffusion; however, we showed that p10.8 is actively imported into the nucleus, because (1) GFPp10.8 and fragments GFPp10.8(1–40) and GFPp10.8(1–65) were able to transport the GFP protein into the nucleus, but this property was not displayed by fragments p10.8 (65–95) and p10.8(40–95); (2) GFP(1–40) could translocate GST to the nucleus, and molecular mass of GFPp10.8(1–40)GST exceeded the limit for free diffusion through NPCs. Our data thus suggest that p10.8 is imported into the nucleus through the NPC via a signal-mediated nuclear localization.

The active import of proteins into nuclei requires NLSs [11-15]. The best-characterized transport sequences of classic NLSs comprise one or two short stretches of basic lysine- or arginine-rich residues. Recently, a variety of nonconforming NLSs that are not particularly rich in lysine or arginine have been identified in various viral and cellular proteins [16-19]. However, in addition to linear NLSs, discontinuous epitopes or a tertiary structure to contribute to the nuclear import of proteins have been described [20-22]. Our study indicate that the nuclear targeting signal, p10.8(1–40), does not contain any stretches of basic residues that typify the classic NLS sequences, even if it is highly conserved among MDRV. Thus, p10.8(1–40) shows characteristics that distinguish it from classic NLSs. Moreover, nuclear target motif experiments demonstrated that three deletion constructs, GFP(1–10), GFP(10–30), and GFP(30–40) are indispensable, suggesting that full sequences or a complex structure of p10.8(1–40) appears to be essential for nuclear import activity. Our finding might be in keeping with the hypothesis, derived from the import of STAT1 and pUL84, that not only non-canonical sequences but complex folding may generate NLSs [21,23]. Report from well-described human ribonucleoprotein A1 import signal, M9 domain, indicate that aromatic amino acids may generate NLSs [24]. Our sequence analysis of p10.8(1–40) revealed that it is rich in aromatic amino acids (Figure 2C). Determination of mutation of these aromatic amino acids may help to identify whether they are responsible for the active nuclear import of the MDRV p10.8 protein. Recently, another non-conventional nuclear transport mechanisms besides the nuclear pore itself have been well described [25]. This might suggest future experiments to identify the exact transport mechanism used by p10.8.

Similar to nuclear import, the export of a protein from the nucleus depends on the presence of a specific signal, nuclear export signal (NES), with a leucine-rich motif [12,13]. Interestingly, inspection of the p10.8 sequence revealed the presence of one leucine-rich motif (33**L**SV**L**AE**L**SD**L**FD**L**AI47) that matches the consensus for leucine-rich export sequences (Figure 2C). It will therefore be of interest to investigate whether this motif constitutes a functional nuclear export sequence and, consequently, whether p10.8 is a nucleocytoplasmic shuttling protein.

The finding that MDRV, which replicates exclusively in the cytoplasm of the infected cell, expresses a nuclear protein was not surprising, since the small nonstructural protein p17 of avian reovirus and the 14-kDa nonstructural protein σ1s of mammalian reovirus have also been shown to accumulate in the nucleus of transfected and infected cells [6,7]. However, a comparative analysis of the deduced amino acid sequences of p10.8, p17, and σ1s revealed no significant similarities in their primary sequences and showed no conserved functional motifs. Yet, the fact that both p10.8 of MDRV and σ1s of mammalian reovirus can localize to the nucleus and cause apoptosis of infected or transfected cells [7,8], suggests that σ1s and p10.8 may be functionally related. Cytoplasmic reovirus infection profoundly affects the host cell nucleus and its functions. In MDRV-infected cells, the p10.8 protein localizes to the nucleus indicate that p10.8 protein may accomplish an important function inside the nucleus during the phase of the viral replication and ultimately influence disease pathogenesis in the infected host.

## Conclusion

In summary, the results of this study provide important information that p10.8 itself has the ability to translocate across the nuclear pore complex and accumulate in the nucleus. This information will highlight the need for nonconventional nuclear transport study.

## Materials and methods

### MDRV infection

MDRV S14 was propagated, purified, and stored as described previously [6]. Duck embryo fibroblasts (DEF) or Vero cells were grown in 6 well plates with coverslips containing DMEM (GIBCO BRL, MD) with 10% fetal calf serum (GIBCO BRL) at 37°C in an incubator supplied with 5% CO2. These cells were inoculated with S14 at a multiplicity of 10 PFU/cell. The infected cultures were processed for immunofluorescence analyses as described below.

### Plasmid constructs and transfections

Full-length p10.8 was amplified by PCR using specific primers, which introduced a 5′*Eco*R I/3′ *Kpn* I or 5′*Pst* I/3′*Sal* I restriction sites for cloning into pcDNA3.1 and pEGFP-C1, respectively. The various fragments pEGFP-p10.8(1–65), pEGFP-p10.8(65–95), pEGFP-p10.8(1–40), pEGFP-p10.8 (40–95), and pEGFP-p10.8 (40–65) were constructed for mapping the NLS of p10.8. The p10.8 gene fragments fused with GST gene were cloned into pEGFP-C1 vector with primers introduce a 5′ *Eco*RI site and a 3′ *Kpn* I site, named GFPp10.8(1–40)-GST, GFPp10.8(1–10)-GST, GFPp10.8(30–40)-GST, and GFPp10.8(10–30)-GST. Proper framing and accuracy of sequences of all DNA constructs were confirmed by DNA sequencing. All primers used in this study and construction details are available upon request (Additional file 1: Table S1). Confluent Vero or DEF cells were transfected with 5 μg of the recombinant plasmids by using the FuGENE HD transfection reagent (Roche Applied Science) and were then immunostained with the mouse anti-p10.8 serum and with DAPI, as described below.

### Immunofluorescence assay

The immunofluorescence assays were carried out as described previously [1]. Briefly, cells grown on coverslips in 6 well plates with or without S14 infection or plasmid transfection were fixed with 3.7% paraformaldehyde (Sigma, St. Luis, MO) dissolved in PBS (phosphate-buffer saline solution, pH 7.6) for 15 min, permeabilized with 0.1% Triton X-100 and 3%–5% bovine serum albumin (BSA) overnight at 4°C, blocked in 3%–5% BSA, and incubated with the mouse anti-p10.8 antiserum overnight at 4°C. After being washed, the cells were incubated with a secondary goat anti-mouse immunoglobulin G conjugate (ZSGB-BIO, Beijing, China) for 1 h at 30°C. The cells were then washed again, and the nuclei were visualized with DAPI stain. The stained cells were viewed by means of Zeiss Axioplan-2 or a confocal LSM 700 (Carl Zeiss) fluorescence microscopy. Images were analyzed using the emission between 436 and 490 nm. Images were processed with Adobe Photoshop (Adobe Systems).

## Abbreviations

MDRV: Muscovy duck reovirus; DEF: Duck embryo fibroblasts; NLS: Nuclear localization signal; hpi: Hours post infection; hpt: Hours post transfection; NPC: Nuclear pore complex.

## Competing interests

The authors declare that they have no competing interests.

## Authors’ contributions

ML and YZ are responsible for the research design and the writing of this manuscript. DCG, NQ, WLSZ, XFB, YLH, QSZ, and JZ performed the plasmids cloning; p10.8 localization detection after virus infection or plasmids transfection. All authors read and approved the final manuscript.

## Supplementary Material

Additional file 1: Table S1 Fragments and primers used in this study. Click here for file
