# Supplementary material for: Muscovy duck reovirus p10.8 protein localizes to the nucleus via a nonconventional nuclear localization signal
Source: Virol J. 2014 Feb 24;11:37. doi: 10.1186/1743-422X-11-37 (PMC4015296; doi:10.1186/1743-422X-11-37)
Supplement: Additional file 1: Table S1 — Fragments and primers used in this study. [file 1743-422X-11-37-S1.doc]

Additional file 1: Table S1 Fragments and primers used in this study

| Fragments Primers |
| --- |
| pCDNA-p10.8 F: 5‘-CGGAATTC^a^AATGGCTGACGCTTTTG-3’  EcoR I  R: 5‘-GGGGTACCCTAGTTAGATCTCGAG-3’ Kpn I  pEGFP-p10.8 F:5’-AACTGCAGAAATGGCTGACGCTTTTG-3’  Pst I  R:5’-GCGTCGACCTAGTTAGATCTCGAG-3’  Sal I  pEGFP-p10.8(1-65) F: 5’-AACTGCAGAAATGGCTGACGCTTTTG-3’ Pst I  R: 5’-GCGTCGACGGCACCACAAACAGGC-3’ Sal I  pEGFP-p10.8(1-40) F: 5’-AACTGCAGAAATGGCTGACGCTTTTG-3’ Pst I  R: 5’-GCGTCGACGGATAATTCAGCTAAGACC-3’ Sal I  pEGFP-p10.8(40-65) F: 5’-AACTGCAGAGTCCGATCTATTTGAC-3’ Pst I  R: 5’-GCGTCGACGGCACCACAAACAGGC-3’ Sal I  pEGFP-p10.8(40-95) F: 5’-AACTGCAGAGTCCGATCTATTTGAC-3’ Pst I  R:5’-GCGTCGACCTAGTTAGATCTCGAG-3’ Sal I  pEGFP-p10.8(65-95) F: 5’-AACTGCAGCCTGGAACGAATAC-3’ Pst I  R:5’-GCGTCGACCTAGTTAGATCTCGAG-3’ Sal I  GFPp10.8(1-40)-GST F:5’-AACTGCAGAAATGGCTGACGCTTTTGAAGTCC-3’ Pst I  R:5’CCTAGTATAGGGGACATGGATAATTCAGCTAAGACCGATAGG3  GST-F:5’GGTCTTAGCTGAATTATCCATGTCCCCTATACTAGGTTATTGGAAAA3’  GST-R:5’-GCGTCGACATCCGATTTTGGAGGATGGTCG-3’ Sal I  GFPp10.8(1-10)-GST F:5’-AACTGCAGAAATGGCTGACGCTTTT-3’ Pst I  R:5’-CCTAGTATAGGGGACATAATATAGTGG-3’  GST-F:5’CCACTATATTATGTCCCCTATACTAGGTTATTGGAAAA3’  GST-R:5’GCGTCGAC ATCCGATTTTGGAGGATGGTCG3’ Sal I  GFPp10.8(30-40)-GST F:5’-AACTGCAGGGCGTGTCCTATCG-3’ Pst I  R:5’-CCTAGTATAGGGGACATGGATAATTCAGC-3’  GSTF:5’TGAATTATCCATGTCCCCTATACTAGGTTATTGGAAAA3’  GSTR:5’-GCGTCGAC ATCCGATTTTGGAGGATGGTCG-3’ Sal I  GFPp10.8(10-30)-GST F:5’-AACTGCAGAAATTGCCGATTGGGCCGACAT Pst I  R:5’-CCTAGTATAGGGGACATCCCGCTAGTCGTCAGGATAT-3’  GSTF:5’ATATCCTGACGACTAGCGGGATGTCCCCTATACTAGGTTATTGGAAAA3’  GSTR:5’-GCGTCGAC ATCCGATTTTGGAGGATGGTCG-3’ Sal I |

^a^ Restrict sites are underlined.
